# 

**Published:** 1923-05

**Authors:** 


**THE DENTAL REGISTER** is published every month by the Samuel A. Crocker Company, 18 W. 7th Street, Cincinnati, O. It is mailed to subscribers during the last week in each month, being a month-end publication. Have copy for current month here by the 15th of each month. Copy received by the 15th of the month goes into that month's number of the Dental Register. Publication and business office, 18 W. 7th Street, Cincinnati, Ohio. Editorial office, 18 W. 7th Street, Cincinnati, Ohio. Address all correspondence to Editor Dental Register, Box 838, Cincinnati, Ohio, c'o The Samuel A. Crocker Company.

Notices of meetings, announcements of interest to the dental profession, and particulars of new ideas and new inventions will be given free publication in reading section. Advertising section is open to all ethical advertisements.

We are not responsible for opinions or statements made by writers whose articles this magazine may publish—nor does the publishing of any article infer in any way that we indorse the views or opinions expressed by any writer whose article we may publish.

### Sal Hepatica

Materially Aids
Local Treatment

IN

PYORRHEA

Samples on Request

Bristol-Myers Co.
NEW YORK

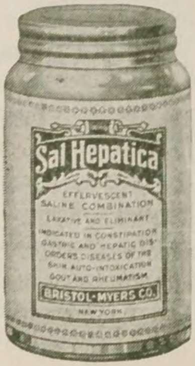

### DIRECTORY

FOR

### Members of the Dental Profession whose practices are limited to a specialty.

The object of this directory is to furnish ready reference to specialists who take referred patients. If you wish your name inserted in this list please correspond with the Dental Register, Box 838, Cincinnati, Ohio.

Your name, specialty, office hours and phone number will be published.

This entire page is reserved for Professional Directory.

### Cincinnati

Dental X-Ray Diagnosis
CHARLES K. FIELD
2709 Woodburn Ave., Walnut Hills
Hours, 10 to 4
Woodburn 2486

Periodontoclasia (Pyorrhea and Oral Prophylaxis)
EDGAR H. EBERLE
605-06 Union Central Bldg., 4th and Vine Sts.
Hours, 9 to 5—Consultation hours by appointment
Main 882

Periodontoclasia (Pyorrhea and Oral Prophylaxis)
MARIE L. BECKERT
25 Groton Bldg., 7th and Race Sts.
Hours, 9 to 5—Consultation hours by appointment
Canal 842

Exodontia (Extraction and Oral Surgery)
ROBERT M. SCHELL
20 W. 9th St., between Vine and Race Sts.
Hours, 1 to 5—Consultation by appointment
Canal 118

### ON TO CLEVELAND—SEPTEMBER 10-14

Dentists, their wives and families, dental dealers and stockholders, manufacturers and everybody interested in the dental profession are looking forward to the great meeting of the American Dental Association in Cleveland next September.

Dentists of Europe are anticipating coming to America to attend this meeting.

Why is the possibility of eight to ten thousand attending this meeting predicted by some?

Because the spirit of the time is one of great progress. The dental profession is riding on the top wave of the true conception of the importance of the teeth in which the laity are deeply interested. Never before has dentistry meant so much to the health of the people. Dentists have recognized this a long time ago but were unable to know and give truly scientific attention to the teeth as we understand it now.

Therefore, the dental profession will have their eyes on the American Dental Association meeting next September. By many an overwhelming attendance is predicted.

Cleveland is an ideal city for the meeting. It has all the facilities including a new modern Auditorium with a seating capacity of over twelve thousand; a wonderful clinic room and every convenience.

Centrally located Cleveland is within five hundred miles of more than half the people of the United States.

The time of the year will insure fine weather, a good time to travel.

The Local Committee have made all arrangements with hotels, so there will be no advanced rates. Everything that can be thought of has been provided for the great meeting. The meeting of 1912 in Cleveland was a great success; this one will be greater.

The program has all been arranged and it shows promise of the greatest *scientific* program that has ever been produced.

Every dentist who can attend this meeting will be abundantly repaid. It would be wise to send for hotel accommodations now.

*Prevent* *Conserve* *Progress*

### ANNOUNCEMENTS

### THE CINCINNATI DENTAL SOCIETY

At the regular meeting of the Cincinnati Dental Society, held Friday, May 18th, 7:30 P. M., at the Literary Club Rooms, the following program was given:

*Essayist:* Dr. Herman Prinz, Philadelphia, Pa., Director of Thomas W. Evans Museum and Dental Institute School of Dentistry, University of Pennsylvania.

*Subject:* "Recent Investigations in Bleaching Teeth."

*Discussion:* Dr. J. Homer Huschart, Dr. H. T. Smith.

Regular dinner was served at 6:30 o'clock.—R. C. Harkrader, Corresponding Secretary.

### MONTANA STATE BOARD OF DENTAL EXAMINERS

The annual meeting of the Montana State Board of Dental Examiners will be held July 9th at Helena, Montana.—Marshall E. Gates, Secretary, Helena, Mont.

### CHICAGO DENTAL SOCIETY

Dr. C. N. Johnson, at a meeting of the Chicago Dental Society, held April 17, 1923, presented the following resolutions which were unanimously adopted:

WHEREAS, the members of the Chicago Dental Society, having learned of the passing of one of its most distinguished and beloved colleagues, Dr. Calvin S. Case, and

WHEREAS, his many years of service to the profession and people in his chosen specialty had made his name synonymous with all that was progressive and scientific in its practice, and

WHEREAS, his sterling character and charming personality had endeared him to an ever-widening circle of friends; therefore, be it

*Resolved*, that the Chicago Dental Society, in session assembled, wishes to place on record its appreciation of his outstanding ability and his many contributions to the science and art of dentistry and his generous helpfulness to those who sought his professional aid; also, be it

*Resolved*, that we give expression to our gratitude for the privilege of having labored with him for so many years and our profound sorrow that he has been taken from our midst.

